# Telemonitoring to improve nutritional status in community-dwelling elderly: design and methods for process and effect evaluation of a non-randomized controlled trial

**DOI:** 10.1186/s12877-018-0973-2

**Published:** 2018-11-16

**Authors:** M. N. van Doorn-van Atten, A. Haveman-Nies, P. Pilichowski, R. Roca, J. H. M. de Vries, C. P. G. M. de Groot

**Affiliations:** 1 Wageningen University & Research, P.O. Box 17, 6700 AA Wageningen, The Netherlands; 2 Habitat&Santé, 373 Chemin Plate-Rousset, F 38330 Biviers, France; 3 Consorci Sanitari de Terrassa, Ctra Torrebonica s/n, E-08227 Terrassa, Spain

**Keywords:** Study protocol, Undernutrition, Prevention, Community-dwelling elderly, Telemonitoring, Real-life setting

## Abstract

**Background:**

A good nutritional status is key for maintaining health and quality of life in older adults. In the Netherlands, 11 to 35% of the community-dwelling elderly are undernourished. Undernutrition or the risk of it should be signalled as soon as possible to be able to intervene at an early stage. However, in the context of an ageing population health care resources are scarce, evoking interest in health enabling technologies such as telemonitoring. This article describes the design of an intervention study focussing at telemonitoring and improving nutritional status of community-dwelling elderly.

**Methods:**

The PhysioDom Home Dietary Intake Monitoring intervention was evaluated using a parallel arm pre-test post-test design including 215 Dutch community-dwelling elderly aged > 65 years. The six-month intervention included nutritional telemonitoring, television messages, and dietary advice by a nurse or a dietician. The control group received usual care. Measurements were performed at baseline, after 4.5 months, and at the end of the study, and included the primary outcome nutritional status and secondary outcomes behavioural determinants, diet quality, appetite, body weight, physical activity, physical functioning, and quality of life. Furthermore, a process evaluation was conducted to provide insight into intervention delivery, feasibility, and acceptability.

**Discussion:**

This study will improve insight into feasibility and effectiveness of telemonitoring of nutritional parameters in community-dwelling elderly. This will provide relevant insights for health care professionals, researchers, and policy makers.

**Trial registration:**

The study was retrospectively registered at Clinical-Trials.gov (identifier NCT03240094) since August 3, 2017.

**Electronic supplementary material:**

The online version of this article (10.1186/s12877-018-0973-2) contains supplementary material, which is available to authorized users.

## Background

A good nutritional status is key for maintaining health and quality of life in older adults [1, 2]. However, in the Netherlands, 11 to 35% of community-dwelling elderly is undernourished. Within this group, the highest percentage of undernutrition is seen among the elderly receiving home care [3]. Considering the negative consequences of undernutrition on morbidity and mortality [4], attention should be given to recognizing undernutrition and the risk of it, so that deterioration can be prevented by timely treatment.

Nutritional screening leads to a better recognition of undernutrition and decreased malnutrition rates in long-term care, and seems to be cost-effective [5, 6]. Although figures are not available for other settings, there is a widespread demand for nutritional screening in at-risk populations [7]. The Dutch undernutrition management guidelines advocate for nutritional screening among community-dwelling older adults [8]. However, compliance to these guidelines is poor: only 16% of home care patients is structurally screened for undernutrition [9]. Furthermore, health care professionals indicate that there is ambiguity concerning screening responsibilities and procedures. They mention that lack of awareness, time, and priority are barriers for nutritional screening among community-dwelling older adults [10].

Concurrently, the increasing burden on health care and focus on self-management of older adults evokes interest in health enabling technologies. eHealth, defined as ‘Health services and information delivered or enhanced through the internet and related technologies’ [11], is viewed as a possibility to meet the needs for cost-effective health care and to improve the access and quality of care [11]. eHealth may be used for nutritional screening in the form of telemonitoring: ‘The use of information technology to monitor patients at a distance’ [12]. Studies have shown that telemonitoring is effective in the management of various chronic diseases [13–15]. To our knowledge, there is only one study in which telemonitoring has been used for monitoring of nutritional parameters in community-dwelling elderly. Results showed that this appeared to be feasible, but due to a small sample size no significant effects could be shown [16].

The PhysioDom Home Dietary Intake Monitoring (HDIM) study focused at telemonitoring and improving nutritional status of community-dwelling elderly with the help of a television based platform and a website for health care professionals. The six-month intervention included telemonitoring of nutritional status, appetite, diet quality, and physical activity. Furthermore, participants received television messages and when necessary dietary advice by a nurse or a dietician. The intervention was implemented in a home care setting and involved participation of community-dwelling elderly, nurses, and dieticians.

Evaluating complex interventions in a real-life setting in which circumstances are less controlled requires an extensive evaluation framework that provides insight into intervention effects, but also into the implementation process and mechanisms of impact [17]. Therefore, this study does not only focus on effect evaluation, but also on evaluation of intervention delivery, feasibility, and acceptability.

This paper aims to describe the design of the PhysioDom HDIM study focusing at nutritional telemonitoring in Dutch community-dwelling older adults in a home care setting. The objectives of the study are: a) to assess the effects of the PhysioDom HDIM intervention on the primary outcome nutritional status and the secondary outcomes behavioural determinants, diet quality, appetite, body weight, physical activity, physical functioning, and quality of life; and b) to assess the implementation process of the telemonitoring intervention including its delivery, feasibility, and acceptability.

## Methods

### Study design

This study ran from February 2016 until June 2017 and followed a parallel arm pre-test post-test design including 215 Dutch participants. The study was carried out in the Netherlands by Wageningen University and care organizations Zorggroep Noordwest-Veluwe and Opella. The study was part of a European project with study sites in the United Kingdom and Spain as well. Each study site employed the same telemonitoring technology, but the exact intervention and the study design varied between study sites to fit the local health care context. This paper therefore only focuses on the study design in the Netherlands. The duration of the intervention was 6 months, preceded by a preparation and recruitment phase. Effect measurements were carried out at the beginning, after 4.5 months, and at the end of the study. Process measurements were carried out throughout the study. The study was retrospectively registered at Clinical-Trials.gov (identifier NCT03240094) since August 3, 2017. The ethics committee of Wageningen University approved the study protocol and all participants gave their written informed consent before the start of the study.

### Study population

The study population consisted of 215 community-dwelling older adults over 65 years receiving home care, informal care, and/or living in a service flat or sheltered accommodation. Individuals were excluded from participation if they were cognitively impaired (Mini Mental State Examination (MMSE) < 20), received terminal care, had cancer, were not able to watch television, or had a physical impairment that prevented them to use the telemonitoring devices properly. The intervention group was recruited in the municipalities of Nunspeet, Harderwijk, Putten, Ermelo, and Renkum; the control group was recruited in the municipalities of Wageningen, Ede, Rhenen, and Veenendaal. Participants were recruited via invitation letters from the care organizations, invitation letters posted in sheltered housing and service flats, and adverts in newspapers and public spaces. After showing an interest in participation, individuals received an information brochure and researchers visited the interested individual at home to answer questions, sign the informed consent, and screen on eligibility criteria.

### Theoretical concept

**Fig. 1:**
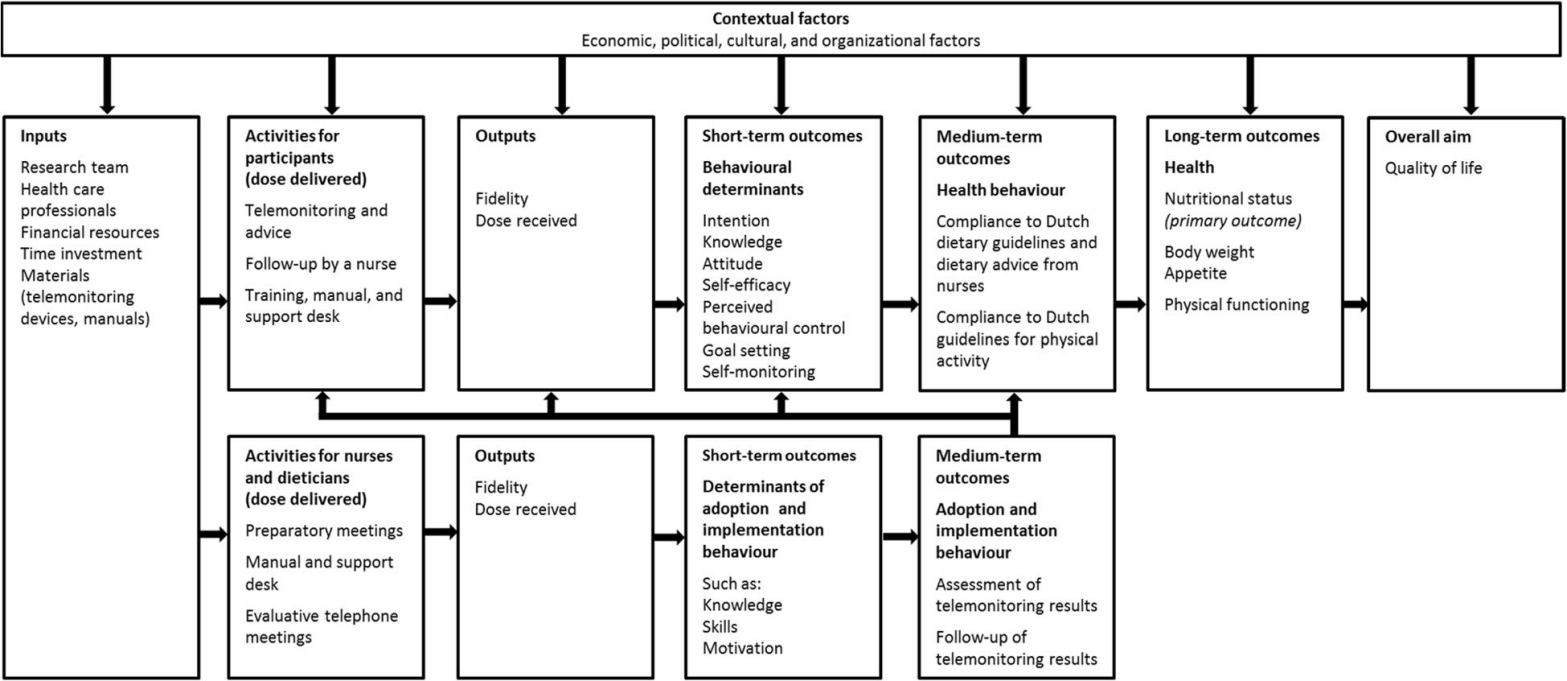
Logic model of the PhysioDom HDIM intervention in the Netherlands

A logic model is useful for planning and evaluating an intervention and visualizes how intervention activities are linked to the hypothesized outcomes on short-term, medium-term and long-term levels [17]. Figure 1 shows the logic model for this study. The logic model guided the selection of the short-term outcomes (intention, knowledge, attitude, self-efficacy, perceived behavioural control, goal setting, self-monitoring), medium-term outcomes (compliance to guidelines for diet and physical activity), and long-term outcomes (nutritional status, physical functioning, and quality of life). Furthermore, the intervention included several behaviour change techniques such as self-monitoring, goalsetting, providing feedback on performance, [18], belief selection, and persuasive communication [19] (Table 1).

**Table 1:** Behaviour change techniques that underpin the PhysioDom HDIM intervention in the Netherlands

Intervention activities	Behaviour change techniques	Definition of behaviour change techniques
Telemonitoring and advice		
Telemonitoring of body weight, nutritional status (MNA-SF), appetite (SNAQ), and blood pressure	Self-monitoring of behavioural outcome	“The person is asked to keep a record of specified measures expected to be influenced by the behaviour change, e.g. blood pressure, blood glucose, weight loss, physical fitness” [18]
Telemonitoring of diet quality (DHD-FFQ) and steps	Self-monitoring of behaviour	“The person is asked to keep a record of specified behaviour/s as a method for changing behaviour” [18]
Setting goals for number of steps and which items of diet quality to improve	Goal setting (behaviour)	“The person is encouraged to make a behavioural resolution (e.g. take more exercise next week). This is directed towards encouraging people to decide to change or maintain change” [18]
Television messages about nutrition and physical activity	Belief selection	“Using messages designed to strengthen positive beliefs, weaken negative beliefs, and introduce new beliefs” [19]
	Consciousness raising	“Providing information, feedback, or confrontation about the causes, consequences, and alternatives for a problem or a problem behaviour” [19]
	Provide information on consequences of behaviour in general	“Information about the relationship between the behaviour and its possible or likely consequences in the general case, usually based on epidemiological data, and not personalised for the individual” [18]
Letters with results of DHD-FFQ and tailored advice on how to improve diet quality and physical activity	Provide feedback on performance	“This involves providing the participant with data about their own recorded behaviour or commenting on a person’s behavioural performance” [18]
Follow-up nurse		
Personal follow-up of nurse in case of risk of undernutrition	Verbal persuasion/persuasive communication	“Guiding individuals and environmental agents toward the adoption of an idea, attitude, or action by using arguments or other means” [19]
Implementation and training		
Manual for participants and health care professionals. For participants: also including cartoons with resistance exercises	Provide instruction on how to perform the behaviour	“Involves telling the person how to perform a behaviour or preparatory behaviours, either verbally or in written form” [18]
Preparatory meetings, workshop, and evaluative telephone meetings with health care professionals	Goal setting (behaviour)	“The person is encouraged to make a behavioural resolution (e.g. take more exercise next week). This is directed towards encouraging people to decide to change or maintain change” [18]
	Action planning	“Involves detailed planning of what the person will do including, as a minimum, when, in which situation and/or where to act” [18]
	Barrier identification/problem solving	“The person is prompted to think about potential barriers and identify ways of overcoming them” [18]
Training for participants	Guide practice	“Prompting individuals to rehearse and repeat the behavior various times, discuss the experience, and provide feedback” [19]
Support desk for participants and health care professionals	Technical assistance	“Providing technical means to achieve desired behavior” [19]

*MNA-SF* Mini Nutritional Assessment-Short Form, *SNAQ* Simplified Nutritional Appetite Questionnaire, *DHD FFQ* Dutch Healthy Diet Food Frequency Questionnaire

### Telemonitoring intervention

#### Telemonitoring measurements and advice

Participants performed several telemonitoring measurements. These measurements should primarily be regarded as intervention components, measurements for research purposes can be found in the next section. Participants measured their body weight weekly and measured their steps 1 week per month. Some participants also measured their blood pressure weekly or bi-weekly upon indication of their nurse. For these measurements, participants received a weighing scale (A&D, type UC-411PBT-C), a pedometer (A&D, type UW-101), and a sphygmomanometer (A&D, type UA-767PBT-CI). Participants received instructions to weigh themselves without heavy clothes and shoes and after voiding. Participants had to measure their blood pressure at a fixed time during the day, while being silent and sitting up straight in a chair with their left arm on the table. Furthermore, participants were asked to fill out questionnaires concerning their nutritional status with the Mini Nutritional Assessment Short-Form (MNA-SF) [20], appetite with the Simplified Nutritional Appetite Questionnaire (SNAQ) [21], and diet quality with the Dutch Healthy Diet Food Frequency Questionnaire (DHD-FFQ) [22]. Participants filled out these questionnaires at the beginning of the study during an interview with the researchers, and 3 months later a second time. Participants could choose how to fill out the questionnaires this second time: via a tablet which they received from the researchers, via their own PC, or via a phone call with the researchers, dependent on the preferences and capabilities of the participants. The results of the telemonitoring measurements were shown on the television of participants. Results from the body weight and blood pressure measurements were sent to the participants’ television by Bluetooth, steps had to be entered manually on the television channel. Furthermore, participants received three short television messages per week with general advice on how to improve nutrition and physical activity. The messages targeted determinants of nutrition and physical activity behaviour such as awareness, knowledge, attitude, and outcome expectations. Participants also received two letters at the beginning and half-way during the study with the results of the DHD-FFQ and customized advice on how to improve diet quality and physical activity.

#### Follow-up by a nurse

Results of the telemonitoring measurements and questionnaires were sent to the project website. On this website, nurses received alerts in case of undernutrition or the risk of undernutrion, obesity or new blood pressure measurements. Alerts for risk of undernutrition were activated if participants lost five to 10 % of baseline body weight in the past 6 months, had an MNA-SF score between eight and 11, and/or had a SNAQ score below 15. Alerts for undernutrition were activated if participants lost more than 10 % of baseline body weight, lost more than 5 % of body weight in the past month, had a body mass index (BMI) below 20 kg/m^2^, and/or had an MNA-SF score of zero to seven. Alerts for obesity were activated if participant had a BMI of 30 kg/m^2^ or higher. Additionally, alerts were activated when participants with heart failure gained two or more kilograms of body weight. The thresholds for alerts were based upon current guidelines and protocols in Dutch health care [8, 23, 24]. In case of risk of undernutrition, undernutrition, obesity, or abnormal blood pressure values, the nurse contacted the participant to provide follow-up. If the participant risked undernutrition, the nurse advised on how to improve protein and energy intake and gave a brochure with advice. If the participant was undernourished, the nurse referred to a GP or dietician. Nurses were aided in processing the alerts by decision trees (Additional file 1) and could consult dieticians from the care organizations if needed.

#### Implementation and training of health care professionals and participants

In the months prior to the intervention, the researchers had four preparatory meetings of one to 2 hours with the nurses and dieticians in which they discussed how implementation could be organized and how the intervention could fit within existing working procedures. During these meetings, nurses and dieticians were trained in using the project website, processing the alerts, and working with the decision trees. Also topics related to change management were covered in the meetings. In the last meeting, a dietician gave a workshop for the nurses with the aim to improve knowledge about nutrition and undernutrition in elderly people. The nurses and dieticians received a manual that covered the information of the preparatory meetings and the workshop. Every one to 2 months, the researchers and nurses held evaluative meetings via telephone to assess implementation and address questions from nurses. At the beginning of the intervention, participants received a training about the use of the television channel, the weighing scale, pedometer, and, if applicable, sphygmomanometer and/or tablet. This training was based on the theory of guided practice [19], took place at the participant’s home and lasted about 45 min. Participants also received a step-by-step illustrated manual. A support desk was available for extra assistance via telephone or at the participant’s home. Furthermore, compliance to the intervention was stimulated through a paper calendar listing the telemonitoring measurements, illustrated cards with positive cues to use the television channel and to adhere to telemonitoring measurements, and three newsletters.

### Participants in the control group receive usual care

#### Research measurements

Research measurements were performed during the screening, at baseline (T0), 4.5 months after baseline (T1), and after 6 months at the end of the intervention (T2). At each time point, trained researchers or research assistants visited the participants at their homes to administer questionnaires in the form of a structured interview or a paper questionnaire and to perform measurements.

During the screening visit, the *background characteristics* age, sex, height, education level, birth country, marital status, living situation (alone or with partner or relatives) and disease history were measured. Items for these characteristics were derived from The Older Persons and Informal Caregivers Survey Minimum DataSet (TOPICS-MDS) [25]. Cognitive functioning was assessed with the MMSE [26]. Furthermore, the presence of dental problems, presence of swallowing problems, type and amount of care or informal care, presence of a diet, and wish for weight reduction were recorded.

The primary outcome *nutritional status* was measured during an interview at T0, T1, and T2 with the Mini Nutritional Assessment (MNA). The MNA consists of 18 items and classifies a person as undernourished, at risk for malnutrition, or normal nutritional status. The outcome is a score ranging from zero to 30, with a higher score indicating a better nutritional status. The MNA is a well-validated tool with high sensitivity, specificity, and reliability [27].

*Behavioural determinants of healthy eating and sufficient physical activity* (defined as eating and being physically active according to Dutch guidelines) were measured at T0, T1, and T2 with a self-developed paper questionnaire. The questionnaire contained 46 statements concerning intention, knowledge, attitude, self-efficacy, perceived behavioural control, goalsetting, and self-monitoring to be answered on a five-point Likert scale, except for the 11 knowledge statements which were answered with true, false, or unsure. Items were derived from validated questionnaires [28–30] or based on previous research [31, 32].

*Diet quality and compliance to physical activity guidelines* were measured with the DHD-FFQ [22]. The Dutch dietary guidelines form the basis of this screener [33]. The DHD-FFQ contains 25 questions and results in a total score ranging from zero to 80, with a higher score meaning better compliance to the dietary guidelines. Eight sub scores ranging from zero to 10 assess compliance to guidelines for vegetables, fruit, fish, alcohol, saturated fatty acids, trans-fatty acids, sodium and dietary fibre. A ninth score assesses compliance to guidelines for physical activity. For this study, compliance to guidelines for protein and vitamin D were additionally assessed. The DHD-FFQ was administered during an interview at T0 and T2. Additionally, participants in the intervention group filled out the DHD-FFQ half-way during the study as intervention component (see intervention section).

*Appetite* was assessed with the SNAQ, a reliable and valid tool for identifying elderly people at risk of unintentional weight loss [21]. The outcome is a score ranging from four to 20, with a higher score indicating more appetite. Appetite was measured during an interview at T0 and T2. In addition to that, participants in the intervention group filled out this questionnaire half-way during the study as intervention component (see intervention section).

*Body weight* was measured with scales from the brand A&D, type UC-411PBT-C at T0, T1, and T2. Participants were weighed without their shoes and heavy clothes.

*Physical functioning* was measured with the Katz-15 questionnaire [34] and the Short Physical Performance Battery (SPPB) [35]. The SPBB test measures balance (three standing positions), gait speed (three meter course), and lower extremity strength (chair stand). The Katz-15 and SPPB were measured at T0 and T2.

*Quality of life* was measured with the Short Form 36 questionnaire (SF-36), including eight dimensions of quality of life: physical functioning, role-physical, bodily pain, general health, vitality, social functioning, role-emotional, and mental health [36, 37]. This questionnaire was filled out on paper at T0, T1 and T2.

Finally, the process evaluation design was guided by the framework of Saunders et al. [38] and included the following process indicators: *recruitment, reach, acceptability, fidelity, dose delivered, dose received, context,* and *applicability* [38–40]. To measure these process indicators, both qualitative and quantitative data were collected using logbooks kept by researchers, questionnaires for participants and health care professionals, and semi-structured interviews with participants and health care professionals. The interviews with participants and health care professionals were guided by a topic list covering questions concerning acceptability of the telemonitoring intervention. Additionally, the participant’s involvement with the television channel (e.g. time, duration, frequency of use) and compliance to telemonitoring measurements were logged automatically by software. These log data provide objective information about the use of the television channel.

### Data – analysis

#### Sample size calculation

The sample size calculation was based on the primary outcome nutritional status. We aimed to detect a difference in MNA change of three and assumed a standard deviation of 6.1 [41]. Assuming an alpha of 0.05, power of 80% and a two-sided test, a sample size of 65 participants per group was required. Taken a drop-out rate of 30% into account, based on Dutch intervention studies in a real-life setting with a similar study population and duration [42–46], we needed 93 participants in each group.

Quantitative data were analysed using SPSS version 22. Continuous data were presented as mean ± standard deviation or standard error of the mean. Categorical data were presented as percentages. Statistical analysis were carried out according to the intention-to-treat principle. Significance was set at *P* < 0.05. We analysed whether data complied to the assumptions required for the analysis methods. Otherwise, transformation of data or non-parametric tests was carried out. Linear mixed models were used to assess differences in changes between the intervention and control group. If necessary, analyses were adjusted for baseline differences between the groups. Qualitative data analysis was carried out using ATLAS.ti (version 7.0).

## Discussion

The aim of this article was to describe the evaluation design of an intervention focusing at improving nutritional status of community-dwelling elderly. To our knowledge, this is the first intervention study that includes telemonitoring of several nutritional outcomes such as diet quality, appetite, and nutritional status including body weight and BMI. Both a process and effect evaluation were included in the study to gain insight into effectiveness, intervention delivery, feasibility, and acceptability.

This study design is expected to provide a thorough evaluation strategy. Firstly, a logic model guided the selection of process indicators and outcome measures at subsequent levels. Secondly, incorporation of behaviour change techniques enables insight into intervention mechanisms [18]. Thirdly, collecting both quantitative and qualitative data provides a complete overview of the process and effects and how these effects could be explained. For example, log data give insight into the participant’s interaction with the television channel so that objective records are available of the time, duration, and frequency of the television channel use. Combining these log data with participant characteristics and results on effect outcomes can be of great value for explaining the effects and unravelling the intervention mechanisms. Furthermore, insight into actual use during implementation provided the opportunity to monitor compliance of participants and to offer additional guidance or training when necessary. Finally, this research is expected to provide durable and broadly relevant results. The telemonitoring technology in this study can become dated, but we also focussed on timeless methodology and principles that underpin the telemonitoring intervention [47]. Examples are the behaviour change techniques to promote a healthy diet and physical activity, and decision trees for health care professionals to decide about follow-up of telemonitoring results.

Concluding, this study is expected to provide valuable insight into feasibility and effectiveness of telemonitoring of nutritional parameters in community-dwelling elderly. This will provide important insights for future development of telemonitoring concepts for the elderly, and how these concepts can be integrated within health care with optimal adoption by the elderly and their health care professionals.

## Additional file

Additional file 1: Decision trees for nurses to follow up on a telemonitoring alert. (DOCX 67 kb)

## Abbreviations

BMI: Body mass index
DHD-FFQ: Dutch healthy diet food frequency questionnaire
MMSE: Mini mental state examination
MNA: Mini nutritional assessment
MNA-SF: Mini nutritional assessment short-form
PhysioDom HDIM: PhysioDom home dietary intake monitoring
SF-36: Short form 36
SNAQ: Simplified nutritional assessment questionnaire
SPPB: Short physical performance battery
