# Supplementary material for: Telemonitoring to improve nutritional status in community-dwelling elderly: design and methods for process and effect evaluation of a non-randomized controlled trial
Source: BMC Geriatr. 2018 Nov 16;18:284. doi: 10.1186/s12877-018-0973-2 (PMC6240290; doi:10.1186/s12877-018-0973-2)
Supplement: Supplementary file 1 — Decision trees for nurses to follow up on a telemonitoring alert. (DOCX 67 kb) [file 12877_2018_973_MOESM1_ESM.docx]

**Additional file 1. Decision trees for nurses to follow up on a telemonitoring alert.**

### *Alerts for risk of undernutrition* The decision tree below describes follow-up by a nurse in case of an alert arising from the lower threshold for weight, lower threshold for BMI, appetite (SNAQ) or nutritional status (MNA-SF).

**YES**

Refer participant to dietician and ask coordination to authorize dietician to get access to telemonitoring results of participant.

Verifiy if dietician is authorized to access telemonitoring results of participant to evaluate progress.

**RISK**

**UNDER-NUTRITION**

Use the telemonitoring results to evaluate nutritional status.

1) Explore causes of risk of undernutrition.

2) Give dietary advice to participant.

**IMPROVEMENT**

**CONCURRENT SYMPTOMS OF DISEASE**

Discuss with dietician if referral to GP is necessary.

**STAGNATION**

Discuss with dietician.

**NO**

**YES**

**NO**

Does the participant already receive treatment from a dietician?

Interpretation by nurse: is there risk of undernutrition or undernutrition? *

Alert from lower threshold for weight, lower threshold for BMI, appetite (SNAQ) or nutritional status (MNA-SF)

Has the nurse already given dietary advice?

How is the progress?

* Is there risk of undernutrition or undernutrition?

| Risk of undernutrition | Undernutrition |
| --- | --- |
| - Between 5 and 10% weight loss in the past half year; *or* - MNA-SF score 8-11; *or* - SNAQ score 0-14. | - More than 10% weight loss in the past half year; or - More than 5% weight loss in the last month; or - BMI below 20; or - MNA-SF score 0-7. |

*Alerts for obesity*

The decision tree below describes the follow-up by a nurse in case of obesity (upper threshold of BMI).

Alert when BMI ≥ 30

**YES**

**NO**

**YES**

**NO**

**YES**

**NO**

Discuss with participant about a referral to a dietician and ask coordination to authorize dietician to get access to telemonitoring results of participant.

No action; keep monitoring.

Based on this consultation, was it decided to refer the participant to a dietician?

Verifiy if dietician is authorized to access telemonitoring results of participant to evaluate progress.

No action; keep monitoring.

Discuss with dietician if weight reduction is desirable*. Is it desirable?

Did the nurse consult the dietician?

***Is weight reduction desirable in case of a BMI of 30 or higher? Advice from Dutch Nutrition center [1]:**

"For the elderly , there are no official cut-off points for overweight and obesity as elderly people who are a little heavier have no greater mortality risk. Older people are advised to lose weight only in case of BMI higher than 30 kg/m2, and only if they have complications or diseases that would benefit from a decrease in body weight, such as type 2 diabetes and cardiovascular diseases. It is important that elderly who want to lose weight do this under the guidance of a dietician. Energy-restricted diets need to be nutrient dense, with a large amount of protein combined with regular exercise . Losing weight is not recommended when energy needs of the elderly is below 1,500 kcal . "

^1^Breedveld, B.P., S. *Ouderen en voeding; Factsheet*. Available from: <http://issuu.com/voedingscentrum/docs/factsheet_ouderen_en_voeding/1?e=1222161/8000342>, accessed 21 August 2017

*Alerts weight increase for heart failure patients*

The decision tree below describes the pathway of care in case of an increase in weight in heart failure patients.

Alert weight increase > 2 kg

Verification: has weight increased with more than 2 kg in the past 3 days?

**NO**

**YES**

Contact GP.

No action, keep monitoring.
